# Cell Reprogramming Strategies for Treating Osteoarthritis and Intervertebral Disc Degeneration

**DOI:** 10.14336/AD.2023.1224

**Published:** 2023-12-24

**Authors:** Zhengfa Jiang, Chen Cao, Yuhao Zhang, Miaoheng Yan, Zongmian Song, Guowei Shang, Hongwei Kou, Hongjian Liu, Yusheng Li, Songfeng Chen

**Affiliations:** ^1^Department of Orthopedics, The First Affiliated Hospital of Zhengzhou University, Zhengzhou, China.; ^2^Department of Orthopedics, Zhengzhou University People’s Hospital, Zhengzhou, China.; ^3^Department of Orthopedics, Henan Provincial People’s Hospital, China.; ^4^Department of Orthopedics, Xiangya Hospital, Central South University, Changsha, Hunan, China.; ^5^National Clinical Research Center for Geriatric Disorders, Xiangya Hospital, Central South University, Changsha, Hunan, China.

**Keywords:** cell reprogramming, intervertebral disc degeneration, osteoarthritis, induced pluripotent stem cells

## Abstract

Osteoarthritis (OA) and intervertebral disc degeneration (IVDD) are the most common degenerative bone and joint diseases, posing a major threat to patients' physical and mental health due to the occurrence of chronic pain and disability. Within this context, the absence of efficacious therapies has led to a growing interest in regenerative medicine. In particular, as a method that can erase the memory of differentiation and re-endow cells with pluripotency, cell reprogramming technologies have ushered in a new era of personalized therapy, which not only show great potential for the treatment of degenerative osteoarthropathies but also promise to achieve tissue regenerative and repair. However, compared to other areas of research, reprogramming technologies to treat OA and IVDD are still in the preliminary stages and require further investigation. This paper briefly introduces the characteristics of cell reprogramming; summarizes the pathological mechanisms of reprogramming to improves energy metabolism, aging, inflammation, oxidative stress, and immune imbalance in OA and IVDD under the background of microenvironment and immunity; highlights the significant advantages of reprogramming-derived cells compared to embryonic stem cells and mesenchymal stem cells, based on these advances, providing important strategies for its development and clinical application in OA and IVDD.

Degenerative bone and joint diseases, represented by osteoarthritis (OA) and intervertebral disc degeneration (IVDD), are increasingly becoming a highly prevalent and serious public health problem as the global aging population increases. The main pathological features of OA and IVDD are decrease in cellular functional activity and imbalance in the synthesis and degradation of extracellular matrix (ECM) within the diseased tissue [1, 2]. Furthermore, complex multi-factorial diseases, oxidative stress, inflammation, immunity, nutrient deprivation, and cell senescence all play key roles in the development of OA and IVDD. To date, highly effective treatments for OA and IVDD are lacking. Although therapeutic strategies such as drugs, physiotherapy, and surgery can relieve some of the clinical symptoms, they remain limited by poor efficacy and a high incidence of adverse events [3, 4].

In order to achieve an in-depth understanding of the pathophysiology of OA and IVDD, increasing interest has been shown in regenerative medicine that restores the initial tissue and metabolic homeostasis of the discs and joints through cellular therapies. Numerous early studies have documented that stem cell therapy can effectively alleviate pain, slow or reverse catabolism, and restore tissue structure [5, 6]. However, both embryonic stem cells and mesenchymal stem cells still face ethical problems, potential immune rejection after transplantation, and tumor formation [7] (Table 1). Within this context, cell reprogramming techniques erase somatic differentiation memory and endow it with pluripotency, which helps overcome previous limitations and promises to achieve better personalized regenerative medicine for OA and IVDD. Furthermore, Ocampo et al. found that cell reprogramming improves cellular senescence characteristics, reverses cellular senescence and extends the lifespan of aging mice, which further shows the great potential of cell reprogramming in treating OA and IVDD [8]. Therefore, cell reprogramming technology shows great potential for age-related OA and IVDD, which can reverse the characteristics of senescent cells, improve the pathological environment of cells, and break the limitations of mesenchymal stem cells or embryonic stem cells, and it is expected to become a groundbreaking treatment strategy, and bring a new hope for OA and IVDD treatments.

**Table 1:** Comparison of different cell sources in cell therapy for OA and IVDD.

Cell source	Advantage	Disadvantage
**Embryonic stem cells**	Totipotent stem cells derived from the fertilized zygote cell; Can differentiate later into ectodermal, mesodermal, and endodermal cells.	Potential immune response; Ethical and moral restriction.
**Mesenchymal stem cells**	Various organizational sources; No moral and ethical restrictions; Multilineage differential potential.	Heterogeneity; Difficulty in differentiation; Poor cartilage regeneration effect;Age-dependent; Chromosomal aberrations and genetic instability.
**iPSC**	Autologous origin; A wide range of sources; No ethical or moral issues.	Genomic instability; Teratoma formation and clonal variations amongst iPSCs derived from the same donor cells; Inefficiency; Heterogeneity; Unable to maintain aging characteristics.
**Direct cell reprogramming**	A wide range of sources; No ethical or moral. issues; No need to go through a pluripotent state; No oncogenic potential; In situ transdifferentiation; Maintain aging characteristics.	The efficiency of reprogramming needs to be improved, and the security needs to be tested.

## Cell reprogramming strategy—iPSC

**Figure 1.**
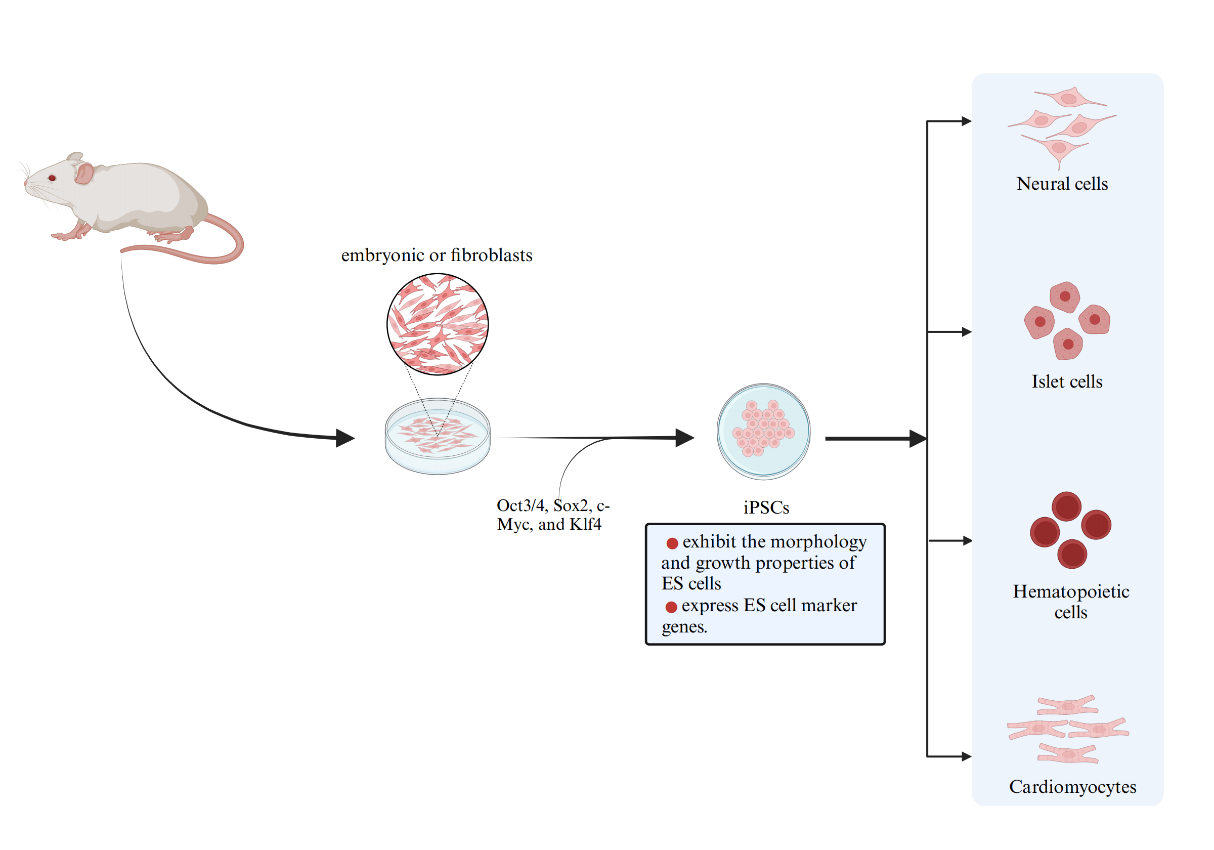
**Detailed process of *in vitro* cell reprogramming strategy (iPSC)**. Mature somatic cells acquired by the body can recover the properties of pluripotent stem cells under the action of various reprogramming factors (Oct 3/4,Sox 2, c-Myc, and Klf 4), and they can be further induced to differentiate into a variety of cells for tissue repair and reconstruction.

The exploration of cell pluripotency began as early as the 18th century, and the success of cloning experiments with clawed frogs and "Dolly" sheep laid an important foundation for the early exploration of somatic cell reprogramming techniques [9, 10]. In 2006, Japanese scientists Takahashi and Yamanaka confirmed that the transfer of four factors (Oct3/4, Sox2, c-Myc, and Klf4) into mouse embryonic or adult fibroblasts was able to reprogram them and produced induced pluripotent stem cells (iPSCs) with similar properties to embryonic stem cells after reprogramming [11] (Fig. 1). The proposal of iPSCs solves the difficult problems of cell source, potential immune rejection after transplantation, and ethical issues in the process of stem cell research, which is a milestone event in the study of related disease models, drug screening, and therapeutic mechanisms [12].

It is well known that OA and IVDD are complex diseases affected by a variety of genetic and environmental factors, and *in vitro* models play an important role in the research of their pathological changes and treatment. However, as the main research method at present, animal models still face numerous challenges in translating to human pathology characterization [13]. In recent years, with the reported transformation of various somatic cells into chondrocytes and nucleus pulposus (NP) cells [14, 15], iPSCs have made remarkable achievements in establishing disease models to explore disease pathological mechanisms, screen drugs, and treat diseases [16, 17]. From the construction of preliminary OA models with typical pathological features from iPSCs, to the interpretation of OA-related genetic variation based on iPSCs' patient-specific characteristics and the evaluation and screen of drugs through the pathological changes of OA models [18-20]. The iPSCs-derived OA model not only overcomes many challenges of the animal model, but also realizes the patient-specific treatment, showing a brighter future. Interestingly, Lapasset et al. have demonstrated that senescent fibroblast-derived iPSCs are able to redifferentiate into fully rejuvenated fibroblasts with telomere lengths, gene expression profiles, oxidative stress levels, and mitochondrial metabolism comparable to those of young fibroblasts, which has important implications for the prevention and treatment of age-related OA and IVDD [21].

## Cell reprogramming strategy—Direct cell reprogramming

**Figure 2.**
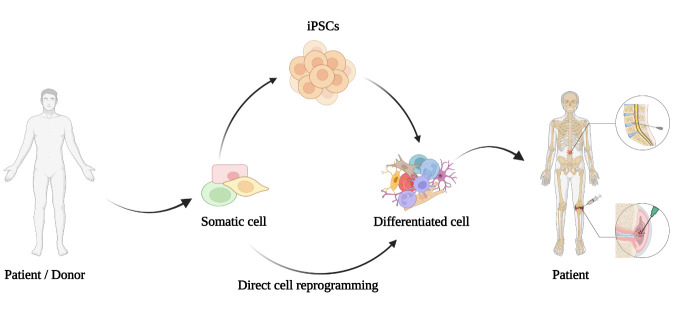
Cell reprogramming strategies for OA and IVDD.

As a "second generation" cell reprogramming strategy, direct cell reprogramming is also known as transdifferentiation. Without altering the genome sequence, it can change the gene expression specific to the original cell lineage through different epigenetic modifications [22]. The main distinction between direct cell reprogramming and iPSC is that direct cell reprogramming can induce in situ the transformation of mature cells into another cell type without transitioning through the pluripotent state (Fig. 2). The transdifferentiation process can produce cells with the same function as iPSCs-derived cells faster and more efficiently and does not show tumorigenicity after transplantation *in vivo*. Presently, Cheng et al. have accomplished the direct reprogramming strategies for the transformation of senescent NP cells to "healthy" NP cells in mice [23]. Moreover, in situ transformation has been proven to avoid the problems of genetic mutations, potential immune rejection, and differentiation uncertainty associated with isolated cell expansion and transplantation in iPSC technology [24]. Although clinical studies of direct reprogramming *in vivo* have not been reported in OA and IVDD, their advantages are self-evident. It is notable that cells differentiated after direct cell reprogramming show clear age-dependent differences in that they still maintain senescence hallmarks such as DNA damage, heterochromatin loss, and nucleocytoplasmic defects, which are in contrast to the erasure of senescence memory by iPSC-derived cells [25]. Therefore, direct cell reprogramming can more stably maintain aging characteristics, which is of great value for the clarification of age-related diseases. To date, despite the reports on direct cell reprogramming related to OA and IVDD are limited, the transformation and characterization of partially mature somatic cells into NP and chondrocytes has been accomplished and the positive effects of direct cell reprogramming in alleviating senescence, inflammatory responses, oxidative stress, cartilage degeneration, and ECM degradation have also been validated.

## Mechanisms of cell reprogramming strategies to treat OA and IVDD

### Cell reprogramming from a cell microenvironmental perspective

Due to the unique anatomical structure of intervertebral discs and joints, their cells are often existed in a relatively hypoxic internal environment and maintain homeostasis through glycolysis as the main pathway, supplemented by mitochondrial metabolism [26, 27]. When cells are exposed to multiple stimuli in and out of the body, enhanced glycolysis accelerates lactate production. The accumulation of large amounts of lactic acid decreases the pH value of the internal environment and exacerbates the hypoxic state of the internal environment, which induces an imbalance between ECM catabolism and anabolism, accelerates cellular senescence and death, and facilitates the occurrence of inflammation and oxidative stress cascade, thus further exacerbating the damage to the tissue microenvironment. In response to this pathological mechanism, many scholars have attempted to improve the intracellular environment by reprogramming multiple pathways of energy metabolism (e.g., nutrient alterations, regulation of rate-limiting enzymes, modulation of inflammation, and epigenetic modifications) to treat OA and IVDD. Wang et al. reprogrammed metabolic pathways by regulating the expression of glucose transporter proteins, effectively enhancing mitochondrial respiration and reducing lactate production [28]. Another report also verified that galactose replacing glucose in human osteoclastic chondrocyte cultures can block inflammatory factor-induced ECM degradation, inhibit the transition of chondrocytes to glycolytic metabolism, and repair impaired mitochondrial dysfunction [29].

In addition, some scholars focus on exploring the inhibition of key enzymes of the glycolytic and mitochondrial respiratory pathways, such as pyruvate kinase M2, hexokinase-2 and pyruvate dehydrogenase kinase 3, which have demonstrated that the modulation of the expression of rate-limiting enzymes are quite effective in decreasing glycolytic metabolism and enhancing mitochondrial respiration, reversing the adverse effects of inflammation, pH reduction, and hypoxia in cells [30, 31]. In order to further clarify the mechanisms of cellular metabolic reprogramming to improve the intracellular environment, emerging studies have revealed that not only the Wnt signaling pathway and NF-κB signaling pathway regulated by the inflammatory factors of TNF-α and IL-1β are able to achieve metabolic transformation, but also the modification level of RNA methylation can regulate the expression of various glycolytic genes, which induces metabolic transformation to glycolysis [30, 32].

### Cell reprogramming from an immune perspective

Immune cells are critical for the onset and progression of OA and IVDD, as they participate in a variety of pathological processes, including cell senescence, oxidative stress, inflammatory responses, and matrix catabolism. Macrophages, a key component of immune cells, can respond to stimuli from different microenvironments to produce polarization types with distinct functions, that is, M1/M2 type macrophages. Polarized macrophages have been highlighted in the progress of OA and IVDD. During the development of OA and IVDD, M1-type macrophage hyperpolarization is an important factor leading to severe tissue injury, obvious inflammatory reaction, nerve/vascular infiltration, and delayed healing. In contrast, M2-type macrophage polarization can effectively reduce a variety of harmful factors, including inflammatory cytokines, matrix metalloproteinases, reactive oxygen species, and NO, and promote tissue repair [33, 34]. Therefore, targeting macrophage polarization is expected to become a new direction for the treatment of OA and IVDD.

Zhou et al. confirmed that restoration of mitochondrial aerobic respiration by regulating intracellular gases guided macrophage polarization towards M2-type macrophages and alleviated inflammatory responses, oxidative stress, ECM degradation, and cartilage degeneration in OA [35]. Recently, Teng et al. reported the relationship between reprogramming and macrophage polarization for the first time in IVDD, confirming that targeted cholesterol metabolism reprogramming is an effective way to regulate macrophage polarization [36]. With the gradual development of macrophage reprogramming in OA and IVDD, the mechanisms and functions of macrophage reprogramming are also under continuous exploration. In addition to the above-mentioned reprogramming of mitochondrial metabolism and cholesterol metabolism, partial microRNAs (miR-224-5p, miR-99b-5p) expression and drugs are also capable of transforming the macrophage polarization phenotype, and the process often involves the inhibition and activation of the NF-κB and MAPK signaling pathways [37, 38].

**Table 2:** *In vivo* study of cell reprogramming for treatment of OA and IVDD.

Study	Models	Result
**Human iPS cell-derived cartilaginous tissue spatially and functionally replaces nucleus pulposus**	Rat	The implantation of human iPSCs-derived cartilaginous tissue into a nuclectomized space of intervertebral disc in nude rats prevented the degeneration of the intervertebral disc and preserved its mechanical properties.
**Human iPSCs can be differentiated into notochordal cells that reduce intervertebral disc degeneration in a porcine model**	Porcine	The notochordal cells differentiated from iPSCs not only showed sustainable phenotype of notochordal cells *in vivo* and *in vitro*, but also showed the functionality of notochordal cells, which played a protective role in IVDD.
**Intradiscal Injection of Induced Pluripotent Stem Cell-Derived Nucleus Pulposus-Like Cell-Seeded Polymeric Microspheres Promotes Rat Disc Regeneration**	Rat	*In vitro* experiments, the expression levels of Col1, Col2, ACAN, and CA12 in NP-like cells differentiated from human iPSCs were significantly increased. *In vivo* experiments, the NP was recovered with increased disc height, water content, and ECM and collagen II synthesis, as well as ordered intervertebral disc structure in the experimental group of injected gelatin microspheres loaded NP-like cells.
**Thermosensitive hydrogels loaded with human-induced pluripotent stem cells overexpressing growth differentiation factor-5 ameliorate intervertebral disc degeneration in rats**	Rat	The results indicate that thermosensitive hydrogel-encapsulated human iPSCs overexpressing the growth differentiation factor-5 gene, while not completely preventing and repairing IVDD, can significantly attenuate the severity of IVDD when compared to the puncture group and the hydrogel group.
**Partial reprogramming strategy for intervertebral disc rejuvenation by activating energy switch**	Mice	The study shows that partial reprogramming through a short-term cyclic expression of Oct-3/4, Sox2, Klf4, and c-Myc rejuvenates phenotypes associated with aging and promotes the redistribution of cytoskeleton organization.
**Nimbolide targeting SIRT1 mitigates intervertebral disc degeneration by reprogramming cholesterol metabolism and inhibiting inflammatory signaling**	Rat	Injecting Nim can increase the expression of SIRT1 in NP tissue, promote the transformation of M1 to M2 macrophages, reduce the loss of ECM in NP, and effectively delay the degeneration of intervertebral discs in rats.
**(-)-Epigallocatechin-3-gallate Ameliorates Intervertebral Disc Degeneration Through Reprogramming of the Circadian Clock**	Rat	They found that EGCG attenuated H2O2-induced ECM degradation in NP cells and inhibited H2O2-induced NP cell apoptosis *in vivo.*
**Non-viral reprogramming of human nucleus pulposus cells with FOXF1 via extracellular vesicle delivery: an in vitro and in vivo study**	Mice	Delivery of FOXF1 via extracellular vesicles can more safely promote the accumulation of glycosaminoglycan, reduce the expression of inflammatory cytokines and matrix-degrading enzymes, and reprograming mouse degenerated NP cells back to a healthy state.
**Fargesin ameliorates osteoarthritis via macrophage reprogramming by downregulating MAPK and NF-κB pathways**	Mice	Fagacin converts macrophage polarization phenotype from M1 to M2 subtype and partially prevents cartilage degeneration by downregulating p38/ERK MAPK and p65/NF-κB signaling.
**LDHA-mediated ROS generation in chondrocytes is a potential therapeutic target for osteoarthritis**	Mice	Their study shows that chondrocytes under inflammatory conditions modulate NF-κB activation, which can lead to reprogramming of cellular metabolism towards glycolysis and lactate dehydrogenase A. Inflammation and metabolism can regulate each other to regulate cartilage degradation.
**Nanomedicines Reprogram Synovial Macrophages by Scavenging Nitric Oxide and Silencing CA9 in Progressive Osteoarthritis**	Mice	Nanomedicines can promote macrophage polarization towards the M2 phenotype, reduce the production of pro-inflammatory cytokines, and induce the expression of cartilage matrix components, thereby effectively protecting cartilage from damage and promoting cartilage repair in the treatment of OA.
**Engraftment of allogeneic iPS cell-derived cartilage organoid in a primate model of articular cartilage defect**	Cynomolgus monkeys	Allogeneic iPSC-derived cartilage organ tissues in chondral defects elicit no immune response, and surviving cartilage tissue cells can integrate as well as remodel into articular cartilage.
**Therapeutic effect of induced pluripotent stem cell -derived extracellular vesicles in an in vitro and in vivo osteoarthritis model**	Rabbit	In a rabbit model, iPSC-derived extracellular vesicles can ameliorate OA lesions with inflammation, subchondral bone protrusion, and articular cartilage destruction, which may involve macrophage polarization to M2 subtypes.
**Repair of cartilage defects in osteoarthritis rats with induced pluripotent stem cell-derived chondrocytes**	Rat	After injection of iPSCs-derived chondrocytes, no immune response was observed in the rat joints, and the gene and protein expression of Col2A1, GAG, and Sox9 were significantly increased, contributing to the improvement of articular cartilage integrity.

## Conclusion and Prospects

OA and IVDD are degenerative musculoskeletal diseases that are highly age-related. With the acceleration of the global aging trend, the prevalence of OA and IVDD and the related socio-economic effects cannot be ignored[39, 40]. Therefore, exploring efficient and comprehensive treatment strategies is particularly urgent. Early studies revealed the great potential of stem cells to improve and repair articular cartilage and degenerated intervertebral discs [41, 42]. In particular, the remarkable therapeutic effect of mesenchymal stem cells in alleviating pain and improving disability in OA and IVDD patients (up to 70%), as well as the successive development of clinical trials, has greatly contributed to the emergence of regenerative repair therapies for OA and IVDD [43-45]. However, cell source, differentiation efficiency, ethics, and potential immune rejection after transplantation are important factors that hinder the development of stem cell therapy. Emerging research displayed that cell reprogramming not only overcomes many of the challenges faced by earlier stem cell therapies but also effectively removes the adverse effects of inflammation, oxidative stress, and cellular senescence, promoting the restoration of intervertebral disc and joint tissue structure and function *in vivo* (Detailed studies are shown in Table 2). Although clinical studies on cell reprogramming to treat OA and IVDD have not been reported, its superior chondrogenic potential and clinical accessibility hold particular promise for developing new treatment strategies for OA and IVDD. Moving ahead, some challenges need to be overcome to effectively transfer the true potential of cell reprogramming to the clinical environment. For clinical research, it is particularly important to identify the optimal cell type, explore efficient cell delivery modalities, and improve the reprogramming efficiency of cells. In addition, identifying genetically defective somatic cells is also critical for reprogramming therapies. Therefore, researchers need to focus more on genetics to deeper understand the effects of genetic mechanisms for reprogramming to treat OA and IVDD. Future interactive applications of gene/base editing, single-cell genomics, 3D printing, and nanomaterial synthesis technologies with cell reprogramming will enable us to avoid limitations such as tumor formation, heterogeneity, differentiation uncertainty, inefficient cell conversion, and genetic variability and to understand the trajectory of cellular differentiation better and develop new therapeutic strategies of OA and IVDD.
